# Bis[2-(1,3-benzothia­zol-2-ylsulfan­yl)eth­yl] ether

**DOI:** 10.1107/S1600536809052301

**Published:** 2009-12-12

**Authors:** Hui-Guo Chen, Xiao-Feng Li, Yan An, Li-Hui Yao, Wei-Sheng Liu

**Affiliations:** a Key Laboratory of Nonferrous Metal Chemistry and Resources Utilization of Gansu Province, College of Chemistry and Chemical Engineering, Lanzhou University, Lanzhou 730000, People’s Republic of China; b Institute of Marine Material and Engineering, Shanghai Maritime University, Shanghai 200135, People’s Republic of China; c College of Chemistry and Chemical Engineering, Lanzhou University, Lanzhou 730000, People’s Republic of China

## Abstract

The complete molecule of title compound, C_18_H_16_N_2_OS_4_, is generated by crystallographic twofold symmetry, with the O atom lying on the rotation axis. The dihedral angle between the ring systems is 80.91 (2)°. In the crystal, adjacent mol­ecules are connected through π–π stacking inter­actions [centroid–centroid distance = 3.882 (2) Å], forming a three-dimensional network.

## Related literature

For coordination polymers in supra­molecular chemistry and crystal engineering, see: Robinson & Zaworotko (1995 ▶); Yaghi & Li (1996 ▶); Fujita *et al.* (1995 ▶); Tong *et al.* (2000 ▶); Bu *et al.* (2003 ▶); Long *et al.* (2004 ▶); Massue *et al.* (2007 ▶); Zou *et al.* (2004 ▶).
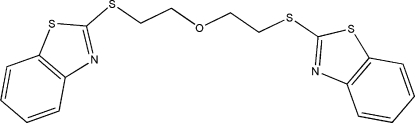


## Experimental

### 

#### Crystal data

C_18_H_16_N_2_OS_4_
*M* *_r_* = 404.57
Monoclinic, 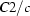

*a* = 24.617 (3) Å
*b* = 4.7085 (3) Å
*c* = 17.7866 (15) Å
β = 116.571 (13)°
*V* = 1843.9 (3) Å^3^
*Z* = 4
Cu *K*α radiation
μ = 4.81 mm^−1^
*T* = 293 K
0.18 × 0.15 × 0.07 mm

#### Data collection

Oxford Diffraction Xcalibur Sapphire3 diffractometer
Absorption correction: multi-scan (*CrysAlis RED*; Oxford Diffraction, 2005 ▶) *T*_min_ = 0.765, *T*_max_ = 1.000
2930 measured reflections
1682 independent reflections
1353 reflections with *I* > 2σ(*I*)
*R*_int_ = 0.020

#### Refinement

*R*[*F* ^2^ > 2σ(*F* ^2^)] = 0.043
*wR*(*F* ^2^) = 0.129
*S* = 1.05
1682 reflections
114 parameters
H-atom parameters constrained
Δρ_max_ = 0.27 e Å^−3^
Δρ_min_ = −0.25 e Å^−3^

### 

Data collection: *CrysAlis CCD* (Oxford Diffraction, 2005 ▶); cell refinement: *CrysAlis CCD*; data reduction: *CrysAlis RED* (Oxford Diffraction, 2005 ▶); program(s) used to solve structure: *SHELXS97* (Sheldrick, 2008 ▶); program(s) used to refine structure: *SHELXL97* (Sheldrick, 2008 ▶); molecular graphics: *SHELXTL* (Sheldrick, 2008 ▶); software used to prepare material for publication: *OLEX2* (Dolomanov *et al.*, 2009 ▶).

## Supplementary Material

Crystal structure: contains datablocks I, global. DOI: 10.1107/S1600536809052301/ng2684sup1.cif

Structure factors: contains datablocks I. DOI: 10.1107/S1600536809052301/ng2684Isup2.hkl

Additional supplementary materials:  crystallographic information; 3D view; checkCIF report

## Figures and Tables

**Fig. 1.**
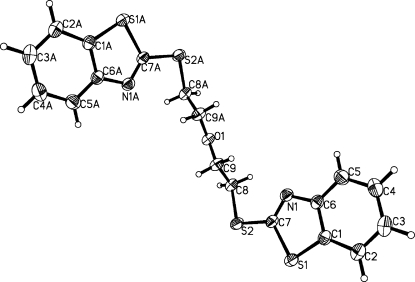
The structure of the title compound, showing the atom-labelling scheme. Displacement ellipsoids are drawn at the 30% probability level. H atoms have been omitted for clarity.

**Fig. 2.**
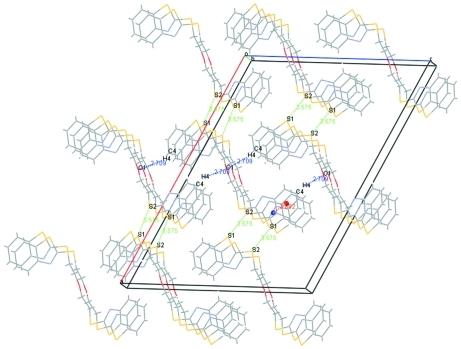
The three-dimensional structure by molecular packing, showing the hydrogen bonds as blue dashed lines [Symmetry codes: (i) x, -y + 1, z + 1/2], S—S bonds as green dashed lines and π-π stacking interactions as red dashed lines.

## supplementary crystallographic information

### Comment

Ligands containing thioether and nitrogenous heterocyclic groups are well established sources for biologically active complexes. In addition, this kind of ligands may form one- or multi-dimensional supramolecular structures *via* the intermolecule interactions such as hydrogen-bond or π-π stacking, attracting intense attention in the field of supramolecular chemistry and crystal engineering (Robinson *et al.*, 1995; Yaghi *et al.*, 1996; Fujita *et al.*, 1995; Tong *et al.*, 2000).

Herein, we report the synthesis and structure of the title compound, namely bis[2-(benzothiazol-2-ylthio)ethyl]ether (Fig.1). As shown in Fig.2, a two-dimensional supramolecular network was formed by hydrogen bonds (Table 2) [Symmetry codes (i): *x*, -*y* + 1, *z* + 1/2] and S—S bonds of 3.575 (2) Å [Symmetry codes (ii): -*x* + 1/2, -*y* + 3/2, -*z* + 2], and there are also weak π-π stacking interactions between the phenyl rings and the thiazolyl rings of adjacent molecules with a centroid-centroid distances of 3.882 Å along *b* direction.

### Experimental

Bis(2-chloroethyl)ether (0.02 mol, 2.86 g) was added dropwise to a hot mixture solution (353 K) of 2-mercaptobenzothiazole (0.04 mol, 6.69 g), KOH (0.04 mol, 2.24 g) in ethanol (100 ml), and the mixture was further stirred at 353 K for 15 h. After cooling, the precipitate was filtered, washed with ethanol and water, and recrystallized from ethanol to obtain white powder. Yield: 56% (Bu *et al.*, 2003; Massue *et al.*, 2007; Long *et al.*, 2004). ^1^H NMR (CDCl_3_, 400 MHz): 3.56 (t, 4H), 3.89 (t, 4H), 7.25 (m, 2H), 7.39 (m, 2H), 7.70 (d, 2H), 7.82 (t, 2H). MS (ESI) m/*z*(%): 405.0 (*M*^+1^).

### Refinement

The H atoms were placed at calculated positions in the riding model approximation (C—H 0.93 Å), with their temperature factors were set to 1.2 times those of the equivalent isotropic temperature factors of the parent atoms.

### Crystal data

**Table:**

C_18_H_16_N_2_OS_4_	*F*(000) = 840
*M**_r_* = 404.57	*D*_x_ = 1.457 Mg m^−^^3^
Monoclinic, *C*2/*c*	Cu *K*α radiation, λ = 1.54178 Å
Hall symbol: -C 2yc	Cell parameters from 1595 reflections
*a* = 24.617 (3) Å	θ = 2.8–68.1°
*b* = 4.7085 (3) Å	µ = 4.81 mm^−^^1^
*c* = 17.7866 (15) Å	*T* = 293 K
β = 116.571 (13)°	Block, colourless
*V* = 1843.9 (3) Å^3^	0.18 × 0.15 × 0.07 mm
*Z* = 4	

### Data collection

**Table:**

Oxford Diffraction Xcalibur Sapphire3 diffractometer	1682 independent reflections
Radiation source: Enhance (Cu) X-ray Source	1353 reflections with *I* > 2σ(*I*)
graphite	*R*_int_ = 0.020
Detector resolution: 16.0855 pixels mm^-1^	θ_max_ = 68.1°, θ_min_ = 2.8°
ω scans	*h* = −29→25
Absorption correction: multi-scan (*CrysAlis RED*; Oxford Diffraction, 2005)	*k* = −5→3
*T*_min_ = 0.765, *T*_max_ = 1.000	*l* = −21→19
2930 measured reflections	

### Refinement

**Table:**

Refinement on *F*^2^	Primary atom site location: structure-invariant direct methods
Least-squares matrix: full	Secondary atom site location: difference Fourier map
*R*[*F*^2^ > 2σ(*F*^2^)] = 0.043	Hydrogen site location: inferred from neighbouring sites
*wR*(*F*^2^) = 0.129	H-atom parameters constrained
*S* = 1.05	*w* = 1/[σ^2^(*F*_o_^2^) + (0.0876*P*)^2^] where *P* = (*F*_o_^2^ + 2*F*_c_^2^)/3
1682 reflections	(Δ/σ)_max_ = 0.001
114 parameters	Δρ_max_ = 0.27 e Å^−^^3^
0 restraints	Δρ_min_ = −0.25 e Å^−^^3^

### Special details

**Table:**

Geometry. All e.s.d.'s (except the e.s.d. in the dihedral angle between two l.s. planes) are estimated using the full covariance matrix. The cell e.s.d.'s are taken into account individually in the estimation of e.s.d.'s in distances, angles and torsion angles; correlations between e.s.d.'s in cell parameters are only used when they are defined by crystal symmetry. An approximate (isotropic) treatment of cell e.s.d.'s is used for estimating e.s.d.'s involving l.s. planes.
Refinement. Refinement of *F*^2^ against ALL reflections. The weighted *R*-factor *wR* and goodness of fit *S* are based on *F*^2^, conventional *R*-factors *R* are based on *F*, with *F* set to zero for negative *F*^2^. The threshold expression of *F*^2^ > σ(*F*^2^) is used only for calculating *R*-factors(gt) *etc*. and is not relevant to the choice of reflections for refinement. *R*-factors based on *F*^2^ are statistically about twice as large as those based on *F*, and *R*- factors based on ALL data will be even larger.

### Fractional atomic coordinates and isotropic or equivalent isotropic displacement parameters (Å^2^)

**Table:**

	*x*	*y*	*z*	*U*_iso_*/*U*_eq_	
C1	0.17154 (13)	0.1689 (5)	1.11310 (16)	0.0505 (6)	
C2	0.18584 (15)	−0.0249 (6)	1.17798 (17)	0.0608 (7)	
H2	0.2256	−0.0860	1.2098	0.073*	
C3	0.13924 (18)	−0.1235 (7)	1.1936 (2)	0.0701 (8)	
H3	0.1474	−0.2568	1.2359	0.084*	
C4	0.08011 (16)	−0.0269 (8)	1.1470 (2)	0.0702 (8)	
H4	0.0494	−0.0954	1.1591	0.084*	
C5	0.06625 (15)	0.1676 (6)	1.0837 (2)	0.0614 (7)	
H5	0.0266	0.2328	1.0534	0.074*	
C6	0.11236 (12)	0.2665 (6)	1.06519 (15)	0.0489 (6)	
C7	0.15597 (12)	0.4948 (6)	0.99910 (14)	0.0483 (6)	
C8	0.09486 (13)	0.8627 (6)	0.86818 (16)	0.0532 (6)	
H8C	0.0791	0.9199	0.9070	0.064*	
H8B	0.0995	1.0323	0.8406	0.064*	
C9	0.05001 (13)	0.6678 (5)	0.80325 (17)	0.0552 (6)	
H9A	0.0683	0.5769	0.7712	0.066*	
H9B	0.0370	0.5215	0.8300	0.066*	
N1	0.10483 (10)	0.4522 (5)	1.00023 (13)	0.0507 (5)	
S1	0.21965 (3)	0.32443 (16)	1.07717 (4)	0.0560 (2)	
S2	0.16852 (3)	0.69930 (17)	0.92661 (4)	0.0592 (3)	
O1	0.0000	0.8363 (5)	0.7500	0.0501 (6)	

### Atomic displacement parameters (Å^2^)

**Table:**

	*U*^11^	*U*^22^	*U*^33^	*U*^12^	*U*^13^	*U*^23^
C1	0.0535 (14)	0.0516 (13)	0.0422 (12)	−0.0026 (11)	0.0176 (11)	−0.0037 (10)
C2	0.0671 (18)	0.0625 (16)	0.0465 (14)	0.0052 (13)	0.0197 (13)	0.0058 (12)
C3	0.094 (2)	0.0659 (17)	0.0546 (16)	−0.0017 (16)	0.0364 (17)	0.0077 (13)
C4	0.081 (2)	0.076 (2)	0.0661 (18)	−0.0114 (16)	0.0442 (17)	−0.0024 (15)
C5	0.0579 (16)	0.0686 (18)	0.0606 (17)	−0.0045 (13)	0.0290 (14)	−0.0061 (13)
C6	0.0530 (15)	0.0504 (13)	0.0395 (12)	−0.0021 (11)	0.0171 (11)	−0.0063 (10)
C7	0.0454 (13)	0.0524 (13)	0.0387 (11)	−0.0023 (10)	0.0112 (10)	−0.0007 (10)
C8	0.0561 (15)	0.0484 (13)	0.0442 (13)	0.0012 (11)	0.0127 (11)	0.0024 (10)
C9	0.0528 (15)	0.0482 (13)	0.0482 (13)	0.0051 (11)	0.0078 (12)	−0.0003 (11)
N1	0.0460 (11)	0.0559 (12)	0.0427 (10)	−0.0030 (9)	0.0132 (9)	−0.0023 (9)
S1	0.0438 (4)	0.0679 (5)	0.0484 (4)	0.0033 (3)	0.0136 (3)	0.0090 (3)
S2	0.0450 (4)	0.0713 (5)	0.0519 (4)	−0.0050 (3)	0.0132 (3)	0.0131 (3)
O1	0.0485 (14)	0.0462 (13)	0.0436 (12)	0.000	0.0099 (11)	0.000

### Geometric parameters (Å, °)

**Table:**

C1—C2	1.387 (4)	C7—N1	1.284 (4)
C1—C6	1.396 (4)	C7—S2	1.744 (3)
C1—S1	1.739 (3)	C7—S1	1.755 (3)
C2—C3	1.376 (5)	C8—C9	1.501 (4)
C2—H2	0.9300	C8—S2	1.810 (3)
C3—C4	1.390 (5)	C8—H8C	0.9700
C3—H3	0.9300	C8—H8B	0.9700
C4—C5	1.372 (5)	C9—O1	1.414 (3)
C4—H4	0.9300	C9—H9A	0.9700
C5—C6	1.395 (4)	C9—H9B	0.9700
C5—H5	0.9300	O1—C9^i^	1.414 (3)
C6—N1	1.394 (4)		
			
C2—C1—C6	122.1 (3)	N1—C7—S1	116.8 (2)
C2—C1—S1	128.4 (2)	S2—C7—S1	116.50 (15)
C6—C1—S1	109.5 (2)	C9—C8—S2	112.65 (19)
C3—C2—C1	117.7 (3)	C9—C8—H8C	109.1
C3—C2—H2	121.1	S2—C8—H8C	109.1
C1—C2—H2	121.1	C9—C8—H8B	109.1
C2—C3—C4	121.0 (3)	S2—C8—H8B	109.1
C2—C3—H3	119.5	H8C—C8—H8B	107.8
C4—C3—H3	119.5	O1—C9—C8	107.0 (2)
C5—C4—C3	121.1 (3)	O1—C9—H9A	110.3
C5—C4—H4	119.4	C8—C9—H9A	110.3
C3—C4—H4	119.4	O1—C9—H9B	110.3
C4—C5—C6	119.1 (3)	C8—C9—H9B	110.3
C4—C5—H5	120.4	H9A—C9—H9B	108.6
C6—C5—H5	120.4	C7—N1—C6	110.1 (2)
N1—C6—C5	125.7 (2)	C1—S1—C7	88.23 (13)
N1—C6—C1	115.3 (2)	C7—S2—C8	101.30 (13)
C5—C6—C1	118.9 (3)	C9—O1—C9^i^	111.7 (3)
N1—C7—S2	126.7 (2)		

Symmetry codes: (i) −*x*, *y*, −*z*+3/2.

### Hydrogen-bond geometry (Å, °)

**Table:**

*D*—H···*A*	*D*—H	H···*A*	*D*···*A*	*D*—H···*A*
C4—H4···O1^ii^	0.93	2.71	3.358 (3)	128

Symmetry codes: (ii) *x*, −*y*+1, *z*+1/2.

## References

[bb1] Bu, X. H., Xie, Y. B., Li, J. R. & Zhang, R. H. (2003). *Inorg. Chem.* **42**, 7422–7430. 10.1021/ic034454j 14606838

[bb2] Dolomanov, O. V., Bourhis, L. J., Gildea, R. J., Howard, J. A. K. & Puschmann, H. (2009). *J. Appl. Cryst.* **42**, 339–341.

[bb3] Fujita, M., Kwon, Y. J., Sasaki, O., Yamaguchi, K. & Ogura, K. (1995). *J. Am. Chem. Soc.* **117**, 7287–7288.

[bb4] Long, D. Q., Li, D. J. & Liu, C. Y. (2004). *Chin. J. Synth. Chem.* **12**, 586–588.

[bb5] Massue, J., Bellec, N., Guerro, M., Bergamini, J. F., Hapiot, P. & Lorcy, D. (2007). *J. Org. Chem.* **72**, 4655–4662. 10.1021/jo0701841 17521197

[bb6] Oxford Diffraction (2005). *CrysAlis CCD* and *CrysAlis RED* Oxford Diffraction Ltd, Abingdon, England.

[bb7] Robinson, F. & Zaworotko, M. J. (1995). *J. Chem. Soc. Chem. Commun.* **23**, 2413–2414.

[bb8] Sheldrick, G. M. (2008). *Acta Cryst.* A**64**, 112–122. 10.1107/S0108767307043930 18156677

[bb9] Tong, M. L., Chen, X. M. & Ng, S. W. (2000). *Inorg. Chem. Commun.* **3**, 436–441.

[bb10] Yaghi, O. M. & Li, H. (1996). *J. Am. Chem. Soc.* **118**, 295–296.

[bb11] Zou, R. Q., Li, J. R., Xie, Y. B., Zhang, R. H. & Bu, X. H. (2004). *Cryst. Growth Des.* **4**, 79–84.

